# Intraoperative Radiation Therapy as a Boost in Soft-Tissue Sarcomas of the Extremity: Long-Term Results of a Retrospective Cohort

**DOI:** 10.1016/j.adro.2026.102097

**Published:** 2026-05-25

**Authors:** Elena Riggenbach, Adam Zabini, Attila Kollár, Frank Michael Klenke, Michael B. Künzler, Christophe Kurze, Radu Olariu, Ioana Lese, Emanuel Stutz, Kristina Lössl, Codruta Ionescu

**Affiliations:** a Department of Radiation Oncology, Inselspital, Bern University Hospital and University of Bern, Bern, Switzerland; b Department of Medical Oncology, Inselspital, Bern University Hospital and University of Bern, Bern, Switzerland; c Department of Orthopaedic Surgery and Traumatology, Inselspital, Bern University Hospital, University of Bern, Bern, Switzerland; d Department of Plastic and Hand Surgery, Inselspital, Bern University Hospital, University of Bern, Bern, Switzerland

## Abstract

**Purpose:**

Intraoperative radiation therapy (IORT) is used as a boost modality for soft-tissue sarcoma (STS). It facilitates the precise delivery of a single tumoricidal dose to areas at high risk for residual microscopic disease while minimizing radiation exposure to adjacent radiosensitive structures. This study reports the long-term outcomes of patients with STS of the extremity at high risk of local recurrence treated with high-dose-rate (HDR)-IORT.

**Methods and Materials:**

A cohort of patients with primary STS of the extremities treated between 2013 and 2019 with an intraoperative boost using HDR-IORT as part of their multimodal treatment was retrospectively reviewed. IORT was applied via a flab applicator (flexible silicone-based surface mold) with a dose of 10 Gy prescribed to 0.5 cm depth. Treatment toxicity and recurrence patterns were systematically monitored. Survival was analyzed via the Kaplan-Meier method.

**Results:**

Fifty-two patients with a median age of 70 years at diagnosis (range, 22-87) were included. The median tumor size was 10 cm (range, 2-36). Twenty patients (38.5%) had prior unplanned resection, and 14 (26.9%) had resection with close margins (ie, <1 mm) at oncological excision. External beam radiation therapy was delivered either neoadjuvantly (59.6%) or adjuvantly (38.5%). Eight patients (15.4%) received chemotherapy. At a median follow-up of 80 months (range, 33-128), 21 patients (40.4%) experienced recurrence. Isolated local failure occurred in 2 patients (3.8%), isolated metastatic failure in 16 patients (30.8%), and synchronous local and metastatic failure in 3 patients (5.8%). At 5 years, the local control and distant control rates were 80.2% and 57.7%, respectively, and the progression-free survival and overall survival rates were 51.9% and 58.0%, respectively.

**Conclusions:**

HDR-IORT boost is a precise and effective technique for dose escalation in primary STS of the extremities, demonstrating favorable local control at long-term follow-up without increasing acute or long-term morbidity in this high-risk patient cohort.

## Background

Radiation therapy (RT) has been an essential modality for the management of soft-tissue sarcomas (STS) of the extremities for many decades.^1^ The incorporation of neoadjuvant or adjuvant RT into the standard surgical approach for tumor resection with wide margins may impact survival only in selected subgroups of patients.^2^, ^3^, ^4^ Nevertheless, its efficacy in enhancing local tumor control, irrespective of the risk profile, has been well established. This facilitates limb and function preservation and reduces surgical morbidity.^2^^,^^5^

Local recurrences in patients with STS present significant challenges in their management, with patients exhibiting risk factors such as high-grade tumors, large tumors, close surgical margins, or unplanned resection demonstrating the greatest risk of local failure.^5^, ^6^, ^7^, ^8^ Dose escalation (ie, boosting) to regions at the highest risk of local recurrence with conventionally fractionated external beam RT (EBRT) has shown conflicting results;^9^ however, it is recommended in the postoperative setting, albeit with a moderate quality of evidence.^10^ The main drawbacks of an EBRT boost are the difficulty in tumor bed localization and the higher integral dose to normal tissue. Intraoperative RT (IORT) offers an alternative boost technique, allowing a smaller high-dose volume owing to its steep dose gradient and precise targeting of the high-risk region under visual guidance, without requiring additional margins for positional uncertainty.^11^^,^^12^ Furthermore, organs at risk, such as nerves or skin, can be safely distanced from the target area, thereby further reducing late morbidity. Although several IORT techniques are in use, most published data on STS of the extremities focus on perioperative brachytherapy^13^, ^14^, ^15^ or electron-based IORT.^16^, ^17^, ^18^, ^19^ High-dose-rate (HDR) brachytherapy IORT (HDR-IORT), a less commonly reported technique, offers distinct dosimetric advantages and allows optimal coverage even for tumor beds with irregular surfaces.^20^, ^21^, ^22^ In contrast to perioperative brachytherapy, where fractionated postoperative loading precludes active displacement of dose-limiting normal tissues during irradiation, HDR-IORT enables intraoperative organ-at-risk displacement alongside a steeper dose gradient than electron-based IORT, resulting in a higher surface dose at the target with rapid dose falloff beyond the reference depth.

In this study, we sought to assess whether HDR-IORT, as a dose-escalation strategy, can achieve high tumor control in unfavorable STS of the extremities.

## Methods and Materials

### Study population

This retrospective cohort study was conducted in accordance with the Strengthening the Reporting of Observational Studies in Epidemiology guidelines.^23^ The medical records of patients with histologically confirmed primary extremity STS treated with conservative surgery and RT, including an HDR-IORT boost, between 2013 and 2019 were reviewed. Patients with distant metastasis at the time of diagnosis were excluded. Data on patient demographics, imaging, pathology, treatment, oncologic outcomes, and toxicities were collected. This study was approved by the regional ethics committee. As this was a retrospective study, the ethics committee waived the requirement for informed consent (Art. 34 HFG), provided no patient had documented an objection to use of their data.

### Treatment

IORT was performed during oncologic resection at our sarcoma specialty center. Indications for IORT included tumors adjacent to critical structures, anticipated close or positive margins, and resection following an unplanned primary excision (ie, “whoops” resection), where the initial surgery was performed without appropriate preoperative planning or suspicion of malignancy. EBRT boost was not routinely used as an alternative boosting strategy at our institution.

**Figure 1:**
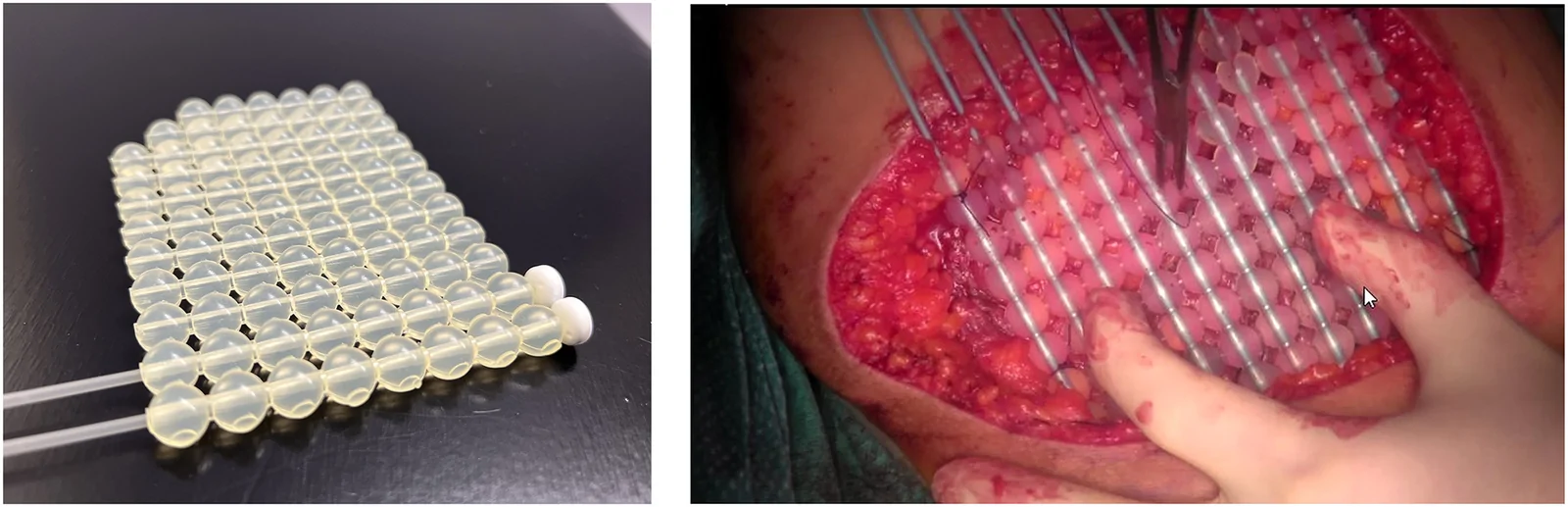
High-dose-rate intraoperative radiation therapy with flab applicator (Freiburg Flap, Elekta): flexible silicone-based surface mold (left) and intraoperative placement on the tumor bed, where sutures may be used to improve conformity and fixation to the resection surface (right).

IORT was delivered via a flab applicator (Freiburg Flap, Elekta; a flexible silicone-based surface mold; Fig. 1) with a dose of 10 Gy prescribed to a depth of 0.5 cm, resulting in an approximate surface dose of 15 to 16 Gy. The applicator was positioned under direct visualization of the tumor bed, targeting areas deemed to have the highest risk of residual tumor or where margins were anticipated to be close, as determined by the surgical team. Conventionally fractionated EBRT (50 Gy in 25 daily fractions) was administered either preoperatively or postoperatively. The use of neo-/adjuvant anthracycline-based chemotherapy was discussed in a shared-decision approach in patients at high risk of distant metastases.

### Patient assessment and follow-up

Local staging was performed via magnetic resonance imaging, and the metastatic workup included a computed tomography scan of the chest. The tumor specimens were reviewed by an expert sarcoma pathologist. Toxicity was documented using the Common Terminology Criteria for Adverse Events 4.03 on a weekly basis during RT and at each follow-up visit. Acute and late toxicities were defined as toxicities occurring within or after 90 days following IORT, respectively.

Major wound complications were defined according to previously established criteria^24^^,^^25^ and subclassified as (1) requiring a secondary operation for wound treatment under general or regional anesthesia, (2) requiring other invasive procedures for wound management, (3) necessitating deep wound packing, or (4) resulting in hospital readmission for wound care.

Follow-up visits, including appropriate imaging (magnetic resonance imaging of the primary site and chest computed tomography) and documentation of late toxicity, were conducted every 3 to 4 months during the first 2 years posttreatment, every 6 months in years 3 to 5, and annually thereafter. The first recurrence was recorded and classified as local (recurrence within the same anatomic compartment as the primary tumor or adjacent to the surgical bed), locoregional (encompassing local recurrence at the primary site and regional nodal disease), or distant (disease at a site anatomically remote from the primary tumor). Local recurrences were further classified relative to the IORT and EBRT target volumes as in-field (recurrence within the planned target volume), marginal (recurrence outside the planned target volume but within the intermediate-to high-dose area receiving 50%-100% of the prescription dose), or out-of-field (recurrence beyond these areas).^26^

### Statistical analysis

The median follow-up time was calculated by excluding deceased patients. Survival probabilities were estimated via the Kaplan-Meier method. Progression-free survival was defined as the time from IORT until tumor recurrence at any site or death by any cause. Overall survival (OS) was defined as the time from IORT until death by any cause. For actuarial local control and distant control, patients were censored for competing events other than local or distant recurrence. All analyses were performed using the R Statistical Software (version 4.2.2; R Foundation for Statistical Computing).^27^

## Results

### Patient and tumor characteristics

Fifty-eight patients received an HDR-IORT boost as part of their multimodal treatment. Of these, 52 patients were included in the analysis. Six patients were excluded: 4 due to metastatic disease at the initial stage, 1 due to non-STS histology, and 1 due to insufficient data. The median age at diagnosis was 70 years (range, 22-87). Tumors had a median size of 10 cm (range, 2-36), with the majority (65.4%) located in the lower extremity. The most common histopathological subtype was undifferentiated pleomorphic sarcoma (42.3%), followed by liposarcoma (17%) (Table 1).

**Table 1:** Patient, tumor, and treatment characteristics (n = 52)

	Median (range) or number (%)
Patient characteristics	
Age at diagnosis (y)	70 (22-87)
Sex	
Women	23 (44.2)
Men	29 (55.8)
Tumor characteristics	
Tumor location	
Upper arm	11 (21.2)
Lower arm	7 (13.5)
Thigh	28 (53.8)
Lower leg	6 (11.5)
Grade	
G1	4 (7.7)
G2	15 (28.8)
G3	32 (61.5)
Unknown	1 (1.9)
Maximum tumor dimension (cm)	10 (2-36)
Tumor stage (TNM eighth edition)	
cT1	5 (9.6)
cT2	17 (32.7)
cT3	16 (30.8)
cT4	6 (11.5)
Unknown	8 (15.4)
Histopathology	
UPS	22 (42.3)
Liposarcoma	9 (17.3)
Myxofibrosarcoma	4 (7.7)
Leiomyosarcoma	4 (7.7)
Synovial sarcoma	4 (7.7)
Other	9 (17.3)
Treatment characteristics	
Resection type	
First resection	
Unplanned excision	20 (38.5)
Oncological resection	32 (61.5)
Final resection	
Marginal resection	31 (59.6)
Wide resection	6 (11.5)
Re-resection	15 (28.8)
EBRT	
Neoadjuvant	31 (59.6)
Adjuvant	20 (38.5)
Not delivered	1 (1.9)

*Abbreviations:* EBRT = external beam radiation therapy; UPS = undifferentiated pleomorphic sarcoma.

Among the 50 patients for whom all necessary parameters for the Sarculator nomogram were available, half were classified as high risk, with an estimated 10-year OS of less than 60%.^28^, ^29^, ^30^

### Treatment characteristics

Twenty patients (38.5%) underwent unplanned resection before referral to our tertiary cancer center and received IORT during the planned reoperation. At oncological excision, 14 of 52 patients (26.9%) had resection with close margins (ie, <1 mm). Overall, 28 patients (53.8%) had either an unplanned initial excision or close resection margins. EBRT (50 Gy in 25 daily fractions) was administered in the neoadjuvant setting in 31 patients (59.6%) and adjuvantly in 20 patients (38.5%) (Table 1). The HDR-IORT boost was 10 Gy prescribed to a depth of 0.5 cm for all patients. Chemotherapy was given to 8 patients (15.4%) (neoadjuvant in 1, adjuvant in 7).

### Oncological outcomes

**Figure 2:**
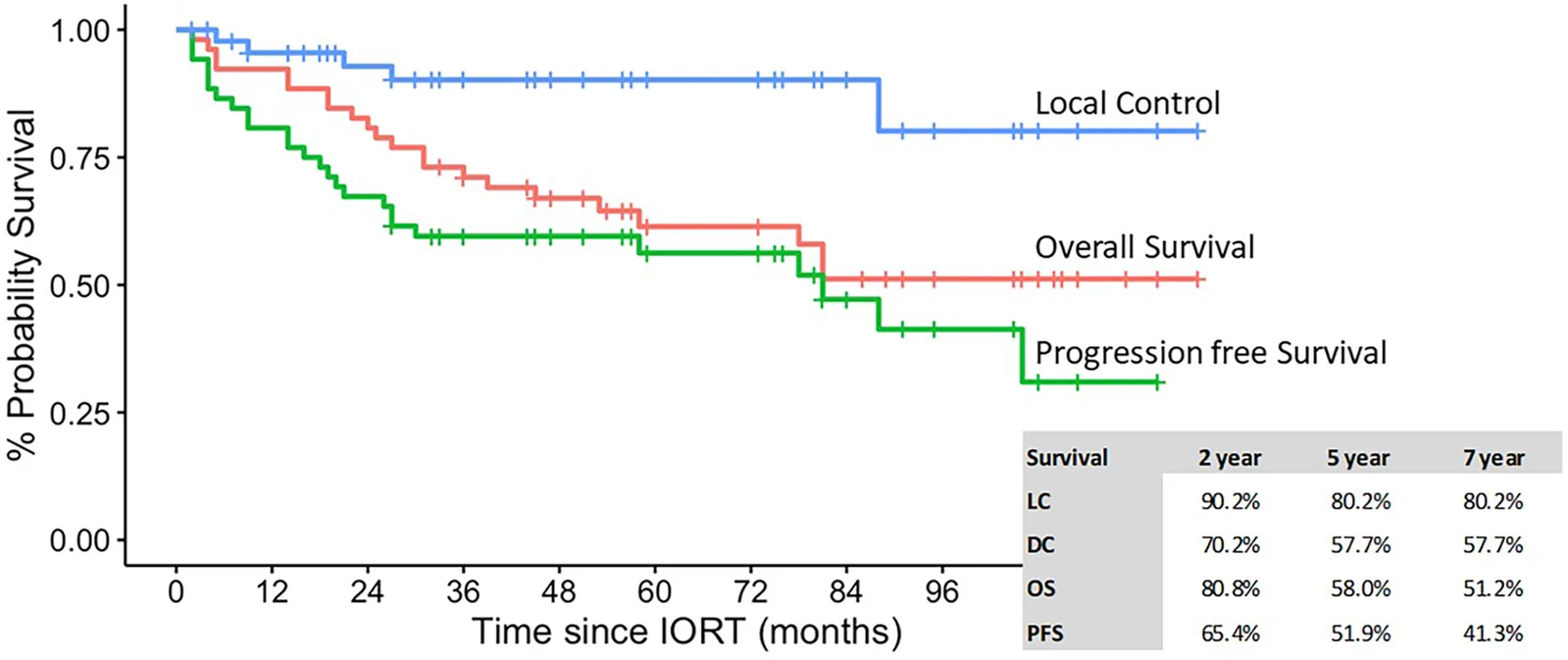
Kaplan-Meier estimates of local control (LC, blue line), overall survival (OS, red line), and progression-free survival (PFS, green line) rates. The tick marks indicate censoring of the data. *Abbreviations*: DC = distant control; IORT = intraoperative radiation therapy.

With a median follow-up of 80 months (range, 33-128), the local control and distant control rates at 2 and 5 years were 90.2% and 80.2%, and 70.2% and 57.7%, respectively (Fig. 2). The 5-year and 7-year progression-free survival rates were 51.9% and 41.3%, respectively, whereas the OS rates were 58.0% at 5 years and 51.2% at 7 years.

Twenty-one patients (40.4%) developed recurrence; 2 patients (3.8%) experienced isolated local failure, 16 patients (30.8%) had isolated metastatic failure, and 3 patients (5.8%) had synchronous local and metastatic failure as their first recurrence. The median time to event was 21 months (range, 5-88) for local recurrences and 14 months (range, 2-106) for distant recurrences, with only 2 cases of late recurrences occurring after 3 years. Local recurrences were either out-of-field (4 of 5) or marginal (1 of 5) in relation to the IORT volume (Table 2). However, 4 of these recurrences were in-field or marginal in relation to the EBRT volume.

**Table 2:** Characteristics of patients with local recurrence

Patient	Age	Sex	Tumor location	Histology	“Whoops”	EBRT	Time to LR (mo)	EBRT-volume	IORT-volume
1	27	men	Thigh	Synovial sarcoma	No	Neoadjuvant	88	Marginal	Out-of-field
2	72	women	Thigh	Spindle cell sarcoma	No	Adjuvant	5	Out-of-field	Out-of-field
3	76	women	Lower arm	UPS	Yes	Adjuvant	9	In-field	Out-of-field
4	71	women	Upper arm	LMS	Yes	Adjuvant	28	In-field	Out-of-field
5	73	women	Thigh	Spindle cell sarcoma	No	Neoadjuvant	21	Marginal	Marginal

*Abbreviations:* EBRT = external beam radiation therapy; IORT = intraoperative radiation therapy; LMS = leiomyosarcoma; LR = local recurrence; “Whoops” = unplanned excision; UPS = undifferentiated pleomorphic sarcoma.

### Adverse effects

Major wound complications occurred in 16 patients (31%) (Table 3). Full wound recovery was achieved in 14 of these patients (93.3%), with only 1 patient developing a chronic nonhealing wound that persisted for several months. One patient with locally advanced disease and extensive regional lymph node metastases died of a postoperative wound infection after a second surgical procedure for regional nodal disease.

**Table 3:** Type and frequency of major wound complications

	Number (%)
Major wound complication	16 (31)
Secondary operation for wound care	13 (25)
Other invasive procedures for wound care	2 (4)
Readmission to hospital for wound care	1 (2)
Deep wound packing	0 (0)
No major wound complication	36 (69)

Severe acute toxicities (Common Terminology Criteria for Adverse Events grade ≥3) were observed in 4 patients (7.7%), whereas the remaining 48 patients (92.3%) experienced only mild or moderate acute toxicities (grade ≤2). Severe late treatment-related toxicities (grade ≥3) were documented in 5 patients: 1 with joint stiffness, 1 with bone fracture, 1 with implant failure, and 2 with late skin/wound complications, including the patient with a persistent nonhealing wound mentioned earlier. Notably, extremity preservation was achieved in all patients during long-term follow-up.

## Discussion

To our knowledge, this study presents the largest series of patients with STS of the extremities treated with an HDR-IORT boost.^21^^,^^22^ Our analysis revealed that despite an unfavorable risk profile, with more than half of the patients having either a close resection margin or having undergone unplanned previous resection, high local control and excellent limb preservation rates were achieved, alongside morbidity outcomes comparable to those seen in other treatment regimens.

Major wound complications, which constitute the most significant acute toxicity combining RT and surgery for STS of the extremities,^24^ occurred in 31% of patients in our cohort. This rate is consistent with the findings of studies that did not incorporate an additional boost.^24^^,^^28^^,^^29^ Notably, these wound complications were transient and were not associated with worse long-term functional outcomes. Similarly, the incidence of other severe acute or late toxicities (grade ≥3) that occurred in approximately 10% of the population was not elevated compared with previously reported rates in the literature.^6^^,^^28^^,^^29^

With advances in diagnostic and therapeutic techniques for STS of the extremities,^30^ local recurrence rates have improved to less than 10% at 3 to 5 years.^6^^,^^28^^,^^31^ However, patients with adverse features such as close surgical margins or unplanned excisions are at significantly increased risk of local recurrence.^7^^,^^32^ They are formally distinct from the patient population without these adverse features and may benefit from local dose escalation. The evidence regarding the role of perioperative or adjuvant boost remains inconclusive and is a matter of debate.^9^^,^^33^^,^^34^

Such a boost can be delivered using EBRT, by perioperative brachytherapy^13^, ^14^, ^15^ or by IORT (typically with electrons or an HDR photon source).^16^, ^17^, ^18^

In the spectrum of brachytherapy-based dose escalation for extremity STS, radioactive sources can be delivered via different techniques and dose rates.^35^ Perioperative HDR brachytherapy is more widely used and involves intraoperative catheter placement followed by fractionated loading over the postoperative days.^13^^,^^14^^,^^36^ Both perioperative and intraoperative approaches permit precise applicator placement under direct visual control. Although perioperative HDR brachytherapy enables staged delivery, single-dose HDR-IORT offers the intraoperative advantage of active displacement of dose-limiting normal tissues from the target area before irradiation. Direct comparison of these strategies with respect to local control and complications remains challenging and has been summarized in a recent review;^35^ results with the more widely studied perioperative brachytherapy approach are encouraging.^36^^,^^37^

The combination of IORT with moderate doses of EBRT consistently achieves excellent local control rates at least equivalent to approaches using EBRT alone, despite being used more often for patients with unfavorable prognostic factors.^13^^,^^14^^,^^17^^,^^18^ In addition, only a few studies have reported long-term results that extend to approximately 7 years. Although the majority of recurrences are known to manifest within the initial 3 years following treatment, our cohort included 2 patients who experienced recurrence after 7 years, with 1 patient presenting with synchronous local and distant events. Long-term follow-up studies are essential to fully understand the efficacy of IORT combined with EBRT in managing STS of the extremities.^38^, ^39^, ^40^

The analysis of local recurrence patterns in our patient cohort revealed that, although 4 of 5 local recurrences occurred within or at the margin of the EBRT target volume, they were outside the IORT volume. This is particularly noteworthy, as IORT was specifically delivered to areas with the highest anticipated risk of recurrence. These findings support the rationale for dose escalation in high-risk areas and suggest that further research on dose-escalation techniques could yield significant improvements in local tumor control.^38^

IORT has the potential to address the 2 inherent limitations of an EBRT boost. It allows for minimal exposure of dose-limiting normal tissues and precise localization of the tumor bed. In addition, delivering a higher dose in a single fraction may offer a tumor-biological advantage for STS, given that the typical α/β ratio (a measure of the fractionation sensitivity of a tissue) for these tumors is estimated to be between 3 and 5.^41^

To our knowledge, no conclusive studies directly comparing a postoperative EBRT boost with HDR-IORT have been published. A retrospective analysis compared 3 other boost modalities—perioperative brachytherapy, intraoperative electrons, and postoperative EBRT—and no boost in extremity STS with positive margins. However, owing to the small sample size and the unequal distribution of patients across these modalities, no definitive conclusions can be drawn.^9^ A frequently referenced study of extremity STS with R1 resections by Al Yami et al^34^ further reported 5-year local recurrence-free survival rates of 90.4% in patients treated with neoadjuvant RT alone versus 73.8% in those receiving an additional 16 Gy postoperative EBRT boost, although these findings remain debated.^33^

Our cohort includes a higher proportion of unplanned excisions, making intertrial comparison challenging. Likewise, an internal comparison within our institution with patients who did not receive an IORT boost would not yield meaningful conclusions, as boost omission was intentionally reserved for patients with inherently more favorable prognostic features.

As no randomized trials have compared different boosting techniques, it remains unclear whether the benefit of a radiation boost depends on its technique.

Given the conflicting evidence regarding the benefits of perioperative chemotherapy for STS, there is no internationally accepted standard for its use.^42^ In recent years, nomograms have been implemented to guide prognosis-based decision-making and patient stratification.^43^^,^^44^ According to the Sarculator tool and the defined threshold of a 10-year predicted OS of 60%, below which the administration of chemotherapy may provide significant benefits,^43^, ^44^, ^45^ approximately half of the patients in our cohort would have been considered to receive chemotherapy, potentially improving outcomes by addressing the predominant distant failure pattern.

This study has several limitations, primarily its retrospective nature and its conduct at a single institution, owing to the limited implementation of this technique. In addition, our data cannot offer a comparison of patients who did not receive an HDR-IORT boost.

From a practical standpoint, although HDR afterloaders are widely available in centers offering brachytherapy and the flexible flab applicator is cost-effective and reusable, an HDR-IORT program typically requires access to a dedicated, shielded operating room, which represents a logistical barrier and likely contributes to challenges in broader dissemination.^46^

## Conclusions

HDR-IORT boost represents a precise, effective, and feasible technique for dose escalation in primary STS of the extremities. This approach does not seem to increase the likelihood of acute or chronic toxicity compared with traditional therapeutic protocols and demonstrates advantageous local control during extended follow-up in this high-risk patient cohort. Institutions with available infrastructure for IORT should engage in collaborative efforts to validate these findings in larger prospective cohorts, thereby justifying the broader use of IORT for STS of the extremities.

## Declaration of AI and AI-Assisted Technologies in the Writing Process

During the preparation of this work the authors used AI models exclusively to provide assistance in the editing and refinement of written content. After using this tool/service, the authors reviewed and edited the content as needed and take full responsibility for the content of the publication.

## Disclosures

None.
